# Alexithymia, Burnout, and Hopelessness in a Large Sample of Healthcare Workers during the Third Wave of COVID-19 in Italy

**DOI:** 10.3390/brainsci13111550

**Published:** 2023-11-05

**Authors:** Domenico De Berardis, Anna Ceci, Emanuela Zenobi, Dosolina Rapacchietta, Manuela Pisanello, Filippo Bozzi, Lia Ginaldi, Viviana Marasco, Maurizio Di Giosia, Maurizio Brucchi, Guendalina Graffigna, Jacopo Santambrogio, Antonio Ventriglio, Marianna Mazza, Giovanni Muttillo

**Affiliations:** 1 Department of Mental Health, Azuenda Sanitaraia Locale 4 Teramo, Contrada Casalena, 64100 Teramo, Italy; 2 Azuenda Sanitaraia Locale 4 Teramo, Circonvallazione Ragusa 1, 64100 Teramo, Italy; anna.ceci@aslteramo.it (A.C.); emanuela.zenobi@aslteramo.it (E.Z.); dosolina.rapacchietta@gmail.com (D.R.); viviana.marasco@aslteramo.it (V.M.); maurizio.digiosia@aslteramo.it (M.D.G.); maurizio.brucchi@aslteramo.it (M.B.); 3 Tutor School of Nursing, University of Milano, 20157 Milan, Italy; manuela.pisanello@asst-fbf-sacco.it; 4 Azienda Socio Sanitaria Territoriale G.Pini-CTO, Via Pini, 9, 20122 Milan, Italy; filippo.bozzi@asst-pini-cto.it (F.B.); giovanni.muttillo@asst-pini-cto.it (G.M.); 5 Department of Life, Health and Environmental Sciences, University of L’Aquila, 67100 L’Aquila, Italy; lia.ginaldi@univaq.it; 6 EngageMinds HUB-Consumer, Food and Health Engagement Research Center, Università Cattolica del Sacro Cuore, 20157 Milan, Italy; guendalina.graffigna@unicatt.it; 7 Azienda Socio Sanitaria Territoriale Brianza, 20812 Limbiate, Italy; j.santambrogio@gmail.com; 8 Department of Clinical and Experimental Medicine, University of Foggia, 71100 Foggia, Italy; a.ventriglio@libero.it; 9 Institute of Psychiatry and Psychology, Department of Geriatrics, Neuroscience and Orthopedics, Fondazione Policlinico Universitario A. Gemelli IRCCS, 00168 Rome, Italy; marianna.mazza@policlinicogemelli.it

**Keywords:** alexithymia, burnout, hopelessness, healthcare workers, HCWs, COVID-19, pandemic

## Abstract

In the present study, we aimed to assess the frequency of and the relationships between alexithymia, burnout, and hopelessness in a large sample of healthcare workers (HCWs) during the third wave of COVID-19 in Italy. Alexithymia was evaluated by the Italian version of the 20-item Toronto Alexithymia Scale (TAS-20) and its subscales Difficulty in Identifying Feelings (DIF), Difficulty in Describing Feelings (DDF), and Externally Oriented Thinking (EOT), burnout was measured with the scales emotional exhaustion (EE), depersonalisation (DP), and personal accomplishment (PA) of the Maslach Burnout Test (MBI), hopelessness was measured using the Beck Hopelessness Scale (BHS), and irritability (IRR), depression (DEP), and anxiety (ANX) were evaluated with the Italian version of the Irritability‚ Depression‚ Anxiety Scale (IDA). This cross-sectional study recruited a sample of 1445 HCWs from a large urban healthcare facility in Italy from 1 May to 31 June 2021. The comparison between individuals that were positive (*n* = 214, 14.8%) or not for alexithymia (*n* = 1231, 85.2%), controlling for age, gender, and working seniority, revealed that positive subjects showed higher scores on BHS, EE, DP IRR, DEP, ANX, DIF, DDF, and EOT and lower on PA than the not positive ones (*p* < 0.001). In the linear regression model, higher working seniority as well as higher EE, IRR, DEP, ANX, and DDF scores and lower PA were associated with higher hopelessness. In conclusion, increased hopelessness was associated with higher burnout and alexithymia. Comprehensive strategies should be implemented to support HCWs’ mental health and mitigate the negative consequences of alexithymia, burnout, and hopelessness.

## 1. Introduction

Alexithymia, which is a personality trait, burnout, and hopelessness are interconnected constructs that might have significantly impacted healthcare workers (HCWs) during the COVID-19 pandemic [1]. HCWs, including doctors, nurses, and other frontline staff, have been facing immense physical and emotional challenges while providing care to an overwhelming number of patients. In this context, it is crucial to understand the implications of alexithymia, burnout, and hopelessness on their mental health and overall well-being [2].

Alexithymia is a relatively stable personality trait, characterised by the difficulty in identifying, describing, and communicating emotion and in distinguishing emotional experiences from underlying physiological activation [3,4]. It also includes a poverty of imaginative processes, externally oriented cognitive style, and conformist social adaptation [5,6]. Peter Sifneos coined the term, which means “no words for mood” to describe a cluster of cognitive and affective characteristics, including “operational thinking”, initially observed among patients with psychosomatic diseases [7]. Several recent studies have demonstrated an important relationship between alexithymia and psychiatric disorders, drug abuse, personality disorders, and various medical diseases [8,9,10,11]. Currently, the frequency of alexithymia ranges between 9 and 23% in the general population, according to several studies [12,13,14,15]. Although it must be said that alexithymia is a little-known disorder in scientific circles, its practical value is undeniable, as it is often associated with higher disease severity, treatment resistance, poor outcomes, and fewer responses to conventional psychotherapies [16,17]. Alexithymic patients show significantly higher levels of anxiety, depression, and general psychological distress [5]. They are more prone to both “functional’’ somatic symptoms and symptoms of emotional turmoil, because they are psychologically poorly equipped [18]. In addition, subjects with alexithymic traits might be more prone to developing suicidal ideation [19,20]. 

HCWs experiencing high stress, trauma, and exhaustion levels during the COVID-19 crisis may find it even more challenging to effectively identify and cope with their emotions [21,22]. The constant exposure to sickness and death and the pressure to make critical decisions can leave HCWs emotionally overwhelmed, further exacerbating their secondary alexithymia [23]. The differentiation between primary or innate alexithymia and secondary alexithymia is based on the theorisation of Freyberger, who described secondary alexithymia with specific reference to a restriction of the capacity for emotional expression and imaginative activity following the presence of acute somatic diseases or psychiatric disorders [24]. Therefore, secondary alexithymia is conceptualised as a defensive strategy based on primitive mechanisms such as denial and projection, which the subject would resort to in order to cope with a traumatic experience such as that of a severe acute and chronic illness [25].

Burnout, from the English word for “burning out”, indicates a specific work situation mainly affecting people engaged in social work [26]. Nurses, psychologists, social workers, doctors (especially those working with chronic or adverse illnesses), teachers, police officers, and judges may experience this emotional exhaustion, leading to decreased professional capacities and severe psychophysical discomfort [27,28]. Therefore, burnout syndrome stems from work-related stress, and it develops if constraints exceed individual resources to cope [29]. Burnout syndrome might arise in poorly managed structures at the organisational level, where there is a poor distribution of workload, low economic remuneration, and high internal conflict [30,31]. People who tend to develop states of anxiety and who experience critical personal and family situations are at greater risk of burnout [32,33].

Burnout has been prevalent among HCWs, particularly during the COVID-19 pandemic [34,35]. The prolonged and intense work demands, including long shifts, higher patient numbers, and the need to constantly adapt to evolving guidelines, have left HCWs at an increased risk of burnout [35]. In the years of the pandemic, doctors, nurses and other health workers have accumulated stress, fear, fatigue, and anxiety, and a great many have turned to the psychological assistance services set up by hospitals and local health authorities [36,37]. Burnout can magnify the challenges of alexithymia, as individuals may struggle to recognise their emotional state, making it harder to address and seek appropriate support [38].

Hopelessness is a psychological construct underlying several psychiatric disorders and refers to the cognitive schemata underlying a negative expectation towards the future [39]. Hopeless subjects believe that nothing will turn out in their favour, that they will never succeed in life, that their important goals will not be achieved, and that their problems will never be solved [40]. This definition of hopelessness corresponds to the third component of the negative triad of Aaron T. Beck’s cognitive model of depression, consisting of a negative view of the self; a negative view of the present; and a pessimistic view of the future [41]. Hopelessness is a psychological construct that can underlie various psychological disorders and related symptoms such as anxiety, depression, suicide, schizophrenia, and substance abuse [42]. In addition, hopelessness has clinical utility for the assessment and prediction of suicide [43].

Hopelessness can be a distressing consequence of the prolonged stress experienced by HCWs during the pandemic [33,44]. The overwhelming number of critically ill patients, limited resources, and witnessing high mortality rates can create a sense of despair and hopelessness. HCWs may feel powerless, with a lack of control over the situation, and may question the impact of their efforts [45,46]. 

As the combined effects of alexithymia, burnout, and hopelessness can compromise HCWs’ mental well-being, potentially leading to feelings of depression, anxiety, and even suicidal ideation, in the present study, the authors aimed to assess the frequency of and the relationships between alexithymia as a personality trait, burnout, and hopelessness in a large sample of HCWs during the third wave of COVID-19 in Italy. In addition, the clinical predictors of hopelessness, as an indirect measure of potential suicidal ideation, were evaluated in this sample. 

## 2. Materials and Methods

### 2.1. Study Design and Participants

This cross-sectional study recruited a convenience sample of 1445 HCWs from a large urban healthcare facility in Italy (province of Teramo, Abruzzo region) that provided care to COVID-19 patients during the pandemic. The average age of HCWs was 44.2 ± 12.1 years, and the average working seniority was 17.7 ± 12.1 years; the number of females was 1046 (72.4%), and the number of males was 399 (27.6%). Recruitment occurred throughout the duration of the study. The self-reported data were collected from 1 May to 31 June 2021, which included the peak of the third-wave pandemic that year in Italy. HCWs were invited through work emails to participate voluntarily without exclusion criteria. The project was directly endorsed and commissioned by the Local Health Authority (Azienda Sanitaria Locale, ASL) of Teramo to evaluate HCWs’ well-being during the pandemic, and therefore, no IRB approval was required. Participants provided their consent online before completing the survey.

The online questionnaire was composed of third sections. The first section showed in detail the steps and aims of the study and ended with a question asking the respondents whether they agreed to participate. The second section included questions about participants’ age (years), sex (male or female), and working seniority.

The third section included six rating scales.

### 2.2. Assessment

Alexithymia was evaluated using the Italian version of the 20-item Toronto Alexithymia Scale (TAS-20) [47]. A score of 61 or higher was considered indicative of alexithymia. The TAS-20 has a three-factor structure: Factor l assesses the capacity to identify feelings and to distinguish between the feelings and bodily sensations of emotional arousal (Difficulty in Identifying Feelings [DIF]); Factor 2 reflects the inability to communicate feelings to other people (Difficulty in Describing Feelings [DDF]); and Factor 3 assesses Externally Oriented Thinking (EOT). This study showed that TAS-20 had good reliability (Cronbach’s alpha for DIF 0.91, DDF 0.90, and EOT 0.75).

A patient’s hopelessness status was measured using the Beck Hopelessness Scale (BHS) [48]. Higher scores on the BHS scale, which ranges from 0 to 20, indicate greater hopelessness. This study showed that BHS had good reliability (Cronbach’s alpha 0.93). 

The presence of burnout was assessed using the Italian version of the Maslach Burnout Inventory (MBI) [49]. The MBI is a tool with 22 items on sentiments related to work, each with a Likert scale of 7 points. On a 7-point Likert scale, respondents rate how frequently they feel certain emotions, from 0 (never) to 6 (every day). Three subscales or aspects are part of the MBI: emotional exhaustion (EE), depersonalisation (DP), and personal accomplishment (PA). High degrees of burnout are correlated with higher item scores in EE and DP and lower scores in PA. This study showed that MBI had good reliability (Cronbach’s alpha 0.92).

The presence of symptoms such as irritability (IRR), depression (DEP), and anxiety (ANX) was evaluated with the Italian version of the Irritability‚ Depression‚ Anxiety Scale (IDA) [50]. This study showed that IRR, DEP, and ANX scales of IDA had good reliability (Cronbach’s alpha range 0.91–0.94).

### 2.3. Statistical Analyses

The differences between subjects that were positive or not positive for alexithymia were tested using analyses of covariance (ANCOVA) with the TAS-20 positivity/negativity as a factor and age, gender, and working seniority as covariates [51]. Effect size calculations were measured as partial eta squared (η^2^). Finally, a block-wise linear regression analysis [52] was conducted to determine which variables were associated with hopelessness (BHS as the dependent variable). In the first block, age, gender, and working seniority were entered. The second block added MBI subscales, IRR, DEP, and ANX to the model. The DIF, DDF, and EOT subscales of TAS-20 were introduced in the last step. The quality of the regression model was also tested using the Durbin–Watson statistic. *p* values ≤ 0.05 were deemed statistically significant. 

## 3. Results

Descriptive statistics of the whole sample and the comparison between subjects, positive or not, for alexithymia are reported in Table 1. The TAS-20 total score was 47.2 ± 12.8; the 15.2% (*n* = 219) of the 1445 HCWs who scored 61 or more were considered positive for alexithymia.

The comparison between individuals that were positive (*n* = 214, 14.8%) or not for alexithymia (*n* = 1231, 85.2%), controlling for age, gender, and working seniority, revealed that positive subjects showed higher scores for BHS, EE, DP, IRR, DEP, ANX, DIF, DDF, and EOT and lower for PA than the not positive ones (*p* range: 0.006–<0.001). The effect size calculation (η^2^) showed that the magnitude of the group effect for all above variables was large.

In the linear regression model (Table 2), a higher working seniority as well as higher EE, IRR, DEP, ANX, and DDF scores and a lower PA score were associated with higher hopelessness (BHS as the dependent variable). In the current analyses, the R^2^ values demonstrated good prediction accuracy, with the model accounting for 42% of the variance in BHS. Also, the Durbin–Watson coefficient was 2.04 (near the optimum of 2.0), and the standardised residuals were normally distributed.

## 4. Discussion

This was the first study investigating the relationships between alexithymia, burnout, and hopelessness in a large sample of HCWs during the Italian third wave of COVID-19. 

Overall, our results demonstrated that the third wave of pandemic emergency had an impact on HCWs’ psychological well-being, which was maybe related to the previous waves and the higher workload due to the care of the COVID-19 patients. Moreover, managing the health emergency linked to the spread of COVID-19 required HCWs to make substantial changes in their work concerning organisational, relational, and safety aspects [53,54]. In several cases, there have been extensions of working hours, increasing demands for on-call duty, activation of extraordinary procedures, a lack of adequate personal protective equipment, and increased physical fatigue [55]. In addition to work-related aspects, during the waves and peaks of the pandemic, there was an increasing difficulty in balancing HCWs’ work and private and family life [56]. Prolonged shifts led HCWs to spend more hours away from their families, and the severity of the situation they had to cope with often made it challenging to adopt adequate recovery strategies [57]. Also contributing to the situation’s complexity were the necessary mobility measures of HCWs in the areas most at risk and the changes due to the reconditioning of entire health facilities, or parts of them, in contexts also wholly dedicated to the COVID-19 emergency [55].

In our study, the frequency of alexithymia in our sample was 15.2%, which is not higher and somewhat in line with that reported in other studies on the general population, despite the pandemic stress [12,13]. We hypothesise that this might be explained by the presence of alexithymic traits before the onset of the pandemic and by the relatively higher numbers of females in our sample as, for women, the frequency rate of clinically relevant alexithymia could be lower [14,58]. 

However, subjects that were positive for alexithymia showed higher levels of hopelessness, elevated burnout in terms of highly enhanced emotional exhaustion and depersonalisation, and low personal accomplishment, together with more distressing irritable, depressive, and anxiety symptoms. These findings are in line with those of previous studies that have demonstrated that subjects with alexithymia often show higher psychological and psychiatric distress than subjects without alexithymia [59,60,61]. In addition, being an HCW with alexithymia and, thus, emotionally poorly equipped, considering the stress of previous and current pandemic waves, may cause emotional dysregulation, leading to the development of a psychological distress [1,62]. 

The finding that the DDF scale of TAS-20 was associated with increased hopelessness and burnout is one of the main results of the present study. To date, no studies have investigated the relationship between burnout and hopelessness, and our study findings might indicate that the development of a state of emotional, physical, and mental exhaustion triggered by excessive stress may have weakened the energy of HCWs, which, in turn, decreased efficiency and left them helpless, hopeless, pessimistic, and angry. Therefore, it is possible to hypothesise that a difficulty in describing feelings might leave the HCWs incapable of communicating personal distress and emotional exhaustion, reducing the possibility of asking for help and receiving it, especially in the case of developing psychiatric symptoms.

Thus, the results of our study may let us hypothesise that the presence of a difficulty in describing feelings is the background on which higher hopelessness and a reduced sense of competence and efficacy, often associated with an increasing negative view of one’s abilities, may develop and might lower HCWs’ both individual and group resilience [63,64]. Alexithymia has been demonstrated to increase the probability of developing burnout, with less professional gratification [65]. As, according to Rutter [66], resilience means the aptitude to cope with life stressors or adversities in a positive way [67], it suggests that positive coping that increases resilience might be lacking in HCWs with alexithymia, who are prone to maladaptive coping styles and an abnormal personal and emotional self-evaluation linked to irritable, anxiety, and depressive symptoms, thus increasing hopelessness and burnout in a vicious circle [1,68]. Nevertheless, individuals with alexithymia might be more suppressive, leading to physiological hyperactivation and health issues, and might struggle to identify their emotions as they are not using effective coping strategies [16,69].

Moreover, alexithymia is a risk factor for experiencing increased levels of chronic psychosocial stress [70]. Chronic psychosocial stress has been exemplified as an imbalance of high demands and a lack of satisfaction with one’s needs [71]. HCWs might be particularly prone to developing maladaptive coping styles that reduce the ability to cope with stressful life events when alexithymia is present [72]. 

In addition, our results may also support Freyberger’s concept of acute “secondary alexithymia” as a reaction to stressful situations [24]. This often occurs following traumatic conditions experienced during critical periods of childhood development or due to strong emotions experienced in adulthood. In addition, secondary alexithymia has recently been related to certain diseases, such as myocardial infarction, hypertension, and rheumatoid arthritis, which suggests that it may also be a coping mechanism [73]. Moreover, acute secondary alexithymia is a temporary state-dependent condition due to distress (in the present study, the third wave of the COVID-19 pandemic in Italy), often decreasing when an acute or subacute stressful situation has finished [74]. In such a perspective, alexithymia during the third wave of the COVID-19 pandemic in Italy has become a coping mechanism protecting the self against emotional distress associated with situations of intense vulnerability [73], and it is possible to hypothesise that higher hopelessness, burnout, and depressive and anxiety symptoms can reflect a state-dependent condition that is related to the persistent and recurrent stressors of the COVID-19 subsequent waves. Nevertheless, this coping strategy is dysfunctional, triggers a vicious circle, and causes the development or aggravation of more severe feelings of hopelessness, increased work exhaustion, and overall psychological distress. 

This may also explain the higher hopelessness, alexithymia, and burnout seen in HCWs with higher working seniority. In terms of socio-demographic variables, some studies have found that younger HCWs with less work seniority show less burnout and psychological distress, as at the beginning of their careers, they are more motivated [75,76], whereas HCWs with more seniority and with a permanent contract suffer more burnout, whether due to monotony, stress, or work overload [77,78]. A good work environment, based on good relations with colleagues, being supported by the organisation, and good remuneration may improve worker motivation even in unfavourable conditions, such as the pandemic, helping to prevent the onset of burnout [79]. In addition, job satisfaction is essential to achieve HCWs who are less prone to burnout and more efficient [80].

Overall, the findings of our study indicate that comprehensive strategies should be implemented to support HCWs’ mental health and mitigate the negative consequences of alexithymia, burnout, and hopelessness [81]. This includes developing interventions that encourage emotional expression and awareness, providing accessible mental health support services, and implementing organisational policies that promote a work–life balance and self-care [82].

This study has several limitations, so the results should be interpreted cautiously. The first limitation was the usage of self-rating scales with probable biases due to the nature of the self-rating scales themselves, with a social desirability bias especially for TAS-20: social desirability bias is a type of response bias that happens when survey participants give answers in accordance with society’s expectations, despite their own beliefs or experiences. In addition, our study lacks follow-up data, as it was cross-sectional. Also, the sample was recruited during the third wave of the COVID-19 pandemic in Italy, and we lack data on previous waves’ effect on HCWs’ psychological well-being; thus, it is not possible to conclude a cause-and-effect relationship. Finally, the sample was constituted mainly of females, which might limit the generalizability of the results concerning gender. 

## 5. Conclusions

In conclusion, HCWs have faced unprecedented challenges during the COVID-19 pandemic, and alexithymia may be relatively prevalent in HCWs during the fourth wave of the pandemic, significantly increasing emotional and mental health consequences. The impact of alexithymia, burnout, and hopelessness cannot be overlooked. Recognising and addressing these psychological constructs is crucial to safeguarding the well-being of HCWs and ensuring the provision of high-quality patient care. Supportive measures, including emotional awareness training, mental health resources, and fostering a positive work environment, are essential for navigating these challenging times and promoting the long-term sustainability of the healthcare workforce.

## Figures and Tables

**Table 1:** Comparison of BHS, MBI scales, TAS-20 scales, IRR, DEP, and ANX among HCWs that were positive (patients with a TAS-20 score ≥ 61, *n* = 214, 14.8%) or negative (patients with a TAS-20 score ≤ 61, *n* = 1231, 85.2%) for alexithymia, controlling for age, gender, and working seniority.

	Overall (*n* = 1445)	Positive for Alexithymia (Score ≥ 61 on TAS-20) (*n* = 219, 15.2%)	Negative for Alexithymia (Score ≤ 61 on TAS-20) (*n* = 1226, 84.4%)	Comparison between Groups (ANCOVA)	Effect Size between Groups (η^2^)
BHS	4.9 ± 4.2	6.3 ± 4.8	4.7 ± 4.0	F = 27.0 df = 1 *p* < 0.001	0.19
EE	18.0 ± 13.3	22.6 ± 15.0	17.1 ± 12.8	F = 30.1 df = 1 *p* < 0.001	0.21
DP	4.8 ± 5.7	6.8 ± 7.3	4.5 ± 5.3	F = 32.4 df = 1 *p* < 0.001	0.22
PA	39.0 ± 8.5	37.6 ± 8.9	39.3 ± 8.4	F = 7.7 df = 1 *p* = 0.006	0.05
DIF	12.3 ± 4.4	14.5 ± 4.8	11.9 ± 4.2	F = 68.8 df = 1 *p* < 0.001	0.46
DDF	15.7 ± 6.2	19.2 ± 7.2	15.1 ± 5.8	F = 80.8 df = 1 *p* < 0.001	0.54
EOT	19.2 ± 4.4	21.0 ± 4.2	18.9 ± 4.3	F = 43.9 df = 1 *p* < 0.001	0.30
IRR	2.7 ± 2.1	3.3 ± 2.2	2.6 ± 2.0	F = 15.9 df = 1 *p* < 0.001	0.11
DEP	5.9 ± 3.2	6.9 ± 3.4	5.7 ± 3.1	F = 24.7 df = 1 *p* < 0.001	0.17
ANX	5.2 ± 2.9	6.3 ± 3.2	5.1 ± 2.8	F = 31.9 df = 1 *p* < 0.001	0.22

**Table 2:** Results of the linear regression analyses with BHS as dependent variable and other variables as independent. Only statistically significant variables are shown.

	Unstandardised Coefficients		Standardised Coefficient	t	*p*	95% Confidence Interval for B	
	B	SE	Beta			Lower Bound	Upper Bound
Working seniority	0.34	0.07	0.10	4.76	<0.001	0.20	0.49
EE	0.21	0.06	0.08	3.60	<0.001	0.10	0.33
PA	−0.08	0.01	−0.17	−7.31	<0.001	−0.11	−0.06
IRR	0.10	0.01	0.05	2.02	0.04	0.03	0.20
DEP	0.34	0.04	0.33	11.08	<0.001	0.35	0.51
ANX	0.20	0.04	0.14	4.55	<0.001	0.12	0.29
DDF	0.65	0.08	0.20	8.10	<0.001	0.49	0.81

R^2^ = 0.42; F = 10.1 df = 1 *p* < 0.001.

## Data Availability

Data available on request due to restrictions, e.g., privacy or ethical.
